# Chemical Synthesis of Atactic Poly-3-hydroxybutyrate (a-P3HB) by Self-Polycondensation: Catalyst Screening and Characterization

**DOI:** 10.3390/polym16121655

**Published:** 2024-06-11

**Authors:** Wael Almustafa, Dirk W. Schubert, Sergiy Grishchuk, Jörg Sebastian, Gregor Grun

**Affiliations:** 1 Department of Applied Logistics and Polymer Sciences, Kaiserslautern University of Applied Science, Schoenstr. 11, 67659 Kaiserslautern, Germany; 2 Institute of Polymer Materials, Department of Materials Science, Faculty of Engineering, Friedrich-Alexander University Erlangen-Nürnberg (FAU), Martensstr. 7, 91058 Erlangen, Germany

**Keywords:** poly-3-hydroxybutyrate, polycondensation, crystallinity, biodegradable, polyester

## Abstract

Poly-3-hydroxybutyrate (P3HB) is a biodegradable polyester produced mainly by bacterial fermentation in an isotactic configuration. Its high crystallinity (about 70%) and brittle behavior have limited the process window and the application of this polymer in different sectors. Atactic poly-3-hydroxybutyrate (a-P3HB) is an amorphous polymer that can be synthesized chemically and blended with the isotactic P3HB to reduce its crystallinity and improve its processability Ring-opening polymerization (ROP) is the most cited synthesis route for this polymer in the literature. In this work, a new synthesis route of a-P3HB by self-polycondensation of racemic ethyl 3-hydroxybutyrate will be demonstrated. Different catalysts were tested regarding their effectiveness, and the reaction parameters were optimized using titanium isopropoxide as the catalyst. The resulting polymers were compared by self-polycondensation for their properties with those of a-P3HB obtained by the ROP and characterized by Fourier transform infrared spectroscopy (FTIR) and gel permeation chromatography (GPC), and the double bond content (DBC) was determined by UV–VIS spectroscopy by using 3-butenoic acid as a standard. Additionally, a life cycle analysis (LCA) of the new method of synthesizing has been carried out to assess the environmental impact of a-P3HB.

## 1. Introduction

The interest of replacing petrochemical polymers with sustainable materials from renewable resources in different applications, like in the automotive and packaging industry as well as in the biomedical section, has increased rapidly in the past few decades. Poly hydroxy alkanoates (PHA) are biodegradable polyesters that can be obtained chemically by polymerization, but also biotechnologically as carbon and energy reserves by a wide variety of microorganisms from different starting materials, which are derived from renewable resources like biomass and organic wastes, but also fatty acids [1].

Poly-3-hydroxybutyrate (P3HB) was first discovered in 1925 by Lemoigne, who described its production in the bacterium Bacillus megaterium, and it is the most common short-chain-length PHA [2]. P3HB is a completely biodegradable polymer which is mainly produced biotechnologically through the fermentation of biomass by different bacteria. This process leads to optically active and therefore absolute isotactic polymers [3]. Isotactic poly-3-hydroxybutyrate (i-P3HB) is resistant to organic solvents and possesses high melting temperatures of 175 °C and excellent mechanical properties, like those of polypropylene (PP) and polyethylene (PE) [4]. However, the high degree of crystallinity, brittleness and fast thermal degradation makes its processing by conventional methods difficult or even impossible, and limits its applications [5]. The introduction of comonomers or blending i-P3HB with other biopolymers can lead to reduced crystallinity by inducing defects in the crystals of i-P3HB and improving its processability [6].

**Figure 1:**
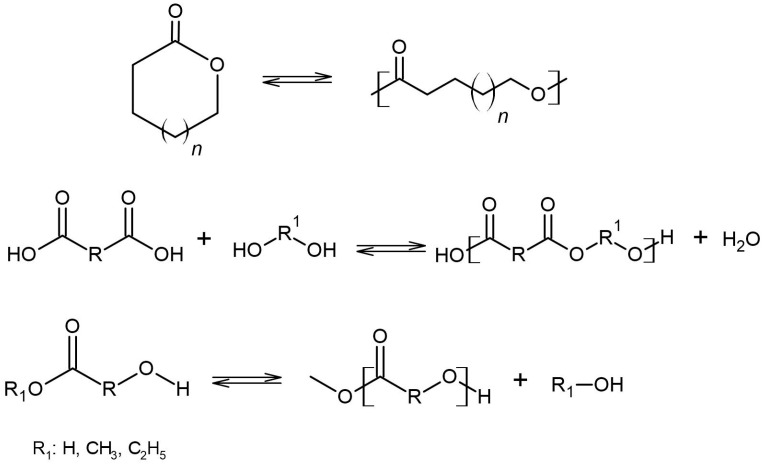
Main polymerization methods of polyesters.

Polyesters can be obtained by different polymerization reactions. These methods are summarized in Figure 1. One of the conventional methods to produce polyesters is the ring-opening polymerization of cyclic esters, which facilitates the obtaining of polymers with high molecular weight (M_w_), narrow polydispersity and defined stereoregularity [7]. However, the cyclic monomers must first be synthesized by a usually complex multi-step synthesis. Polyesters can be also produced by the polycondensation of diacid or diesters with polyols. While there is a wide range of available monomers that can be used for polycondensation reactions, this process usually requires very harsh polymerization conditions [7]. Besides the ring-opening polymerization and the polycondensation, polyesters can be also obtained by the self-polycondensation of hydroxy acids or hydroxy acid esters [8,9,10].

**Figure 2:**
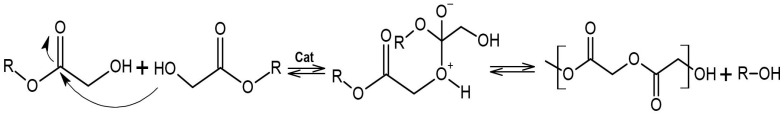
Mechanism of Fischer esterification reaction.

The main mechanism behind the self-polycondensation of hydroxy acids and their esters is the Fischer esterification reaction, which involves the use of a catalyst to facilitate the reaction between the hydroxy acids or their esters (Figure 2) [11].

**Figure 3:**
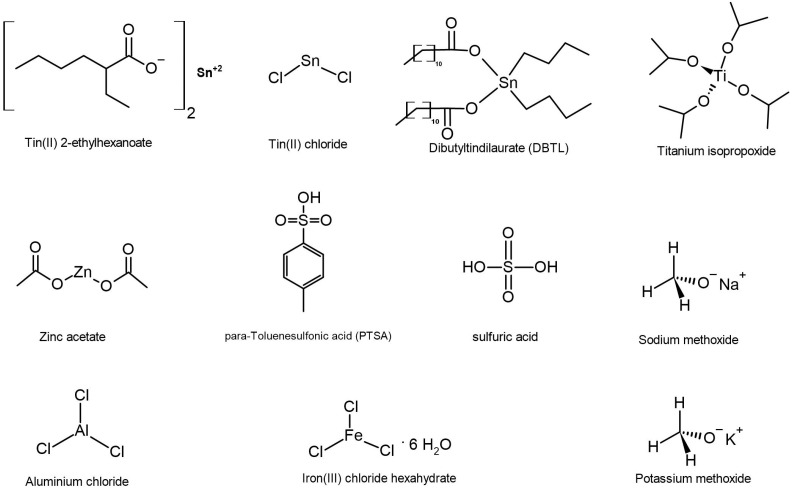
Chemical structure of tested catalysts for self-polycondensation.

This reaction occurs when the carbonyl group of a hydroxy acid or ester is attacked by the nucleophilic hydroxy group of another hydroxy acid or ester, resulting in the formation of an intermediate that produces a distillate, such as water or alcohol. In order to have a high reaction rate, it is important to remove the distillate, which can be achieved by means of a high vacuum and elevated temperature. The success of this reaction is also strongly influenced by the type of monomer and catalyst used. Catalysts can range from acids, such as sulfuric acid and p-toluene sulfonic acid (PTSA), to metals and enzymes, with metal catalysts being particularly desirable due to their high reactivity and thermal stability, making them ideal for producing high molecular weight polymers by self-polycondensation [12,13]. Figure 3 shows the chemical structure of the catalysts which have been tested in this work. Tin catalysts, as well as zinc acetate, have been reported to be effective in the polymerization of lactic acid and glycolic acid, resulting in high molecular weight polymers with controlled properties [14,15]. Moreover, titanium isopropoxide and iron chloride have also been shown to promote polycondensation by increasing the electrophilic character of the carbonyl group of an ester, resulting in polyesters with molecular weights up to 2 × 10^4^ g/mol, such as the reaction product of methyl 4-4hydroxytetrahydrofuran-2-carboxylate with linked tetrahydrofuran rings [16,17].

**Figure 4:**
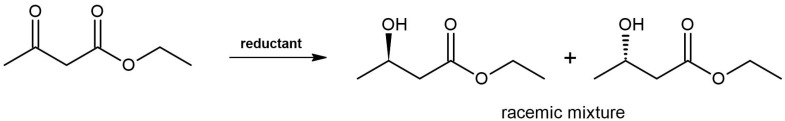
Synthesis of ethyl 3-hydroxybutyrate with sodium borohydride.

While the chemical synthesis of polyhydroxyalkanoates can produce polymers with certain properties that cannot yet be achieved by biotechnology, it is more expensive and laborious than biotechnological production. The racemic ethyl 3-hydroxybutyrate, which can be used as starting material for the synthesis of a-P3HB, can be easily prepared by the reduction of the cheap and commercially available ethyl acetoacetate. In this reaction, different reductants can be used, such as the chiral reductant baker’s yeast or the achiral reductants lithium aluminum hydride (LiAlH_4_) or sodium borohydride (NaBH_4_). However, only the achiral reductants yield a racemic mixture. (Figure 4) [18,19,20]. Thus, the chemical synthesis of a-P3HB can be carried out without high costs, which makes scaleup trails more sustainable.

A further aspect that warrants discussion is the environmental assessment of the chemical synthesis of a-P3HB. To fully assess the sustainability of any synthesis, a comprehensive life cycle assessment is crucial. This methodology enables the examination of multiple factors, including energy consumption and carbon dioxide (CO_2_) emissions. By using a life cycle assessment, researchers can obtain valuable insights into the environmental impact associated with each stage of the chemical synthesis. This includes not only the raw material extraction and production phases but also the utilization, transportation, and disposal phases.

## 2. Materials and Methods

Racemic ethyl 3-hydroxybutyrate, β-butyrolactone, ethyl acetoacetate, sodium borohydride, dichloromethane, dibutyltin dilaurate (DBTL), tin-3-chloride, zinc acetate, sulfuric acid (1 mol/L), iron chloride hexahydrate, aluminum chloride, sodium methanolate, and para-toluene sulfonic acid were purchased from Sigma Aldrich (Darmstadt, Germany). Tin (II) 2-ethylhexanoate was supplied by Evonik (Essen, Germany). The vacuum pump PC 3001-VARIO select from Vacuubrand (Wertheim, Germany) was used to regulate the vacuum.

### 2.1. Synthesis of Racemic Ethyl-3-hydroxybutyrate

Racemic ethyl 3-hydroxybutyrate was synthesized as described in reference [20]. The reaction mechanism is illustrated in Figure 4. Therefore, in a 100-mL round-bottomed flask, sodium borohydride (1.5 g, 40 mmol) was added to 25 mL of ethanol, and the mixture was cooled to 0 °C using an ice bath. Subsequently, a solution of ethyl acetoacetate (5.0 g, 38 mmol) in 15 mL of ethanol was added, and the resulting solution was stirred at 0 °C for 15 min. The mixture was then allowed to warm to room temperature and stirred for an additional 15 min. The solvents were evaporated on a rotary evaporator, and the resulting white solid was suspended in 30 mL of dichloromethane. While cooling in an ice bath, 30 mL of 1 M hydrochloric acid was added dropwise, while stirring. The organic layer was separated, and the aqueous layer was extracted twice with 20 mL portions of dichloromethane. The organic layers were combined, dried using magnesium sulfate, and then filtered. The solvent is evaporated using a rotary evaporator with a water bath temperature at room temperature. The product was characterized using FTIR and the refractive index was used to determine it.

### 2.2. Synthesis of Atactic P3HB by Self-Polycondensation from Racemic Precursor and by Ring-Opening Polymerization

**Figure 5:**
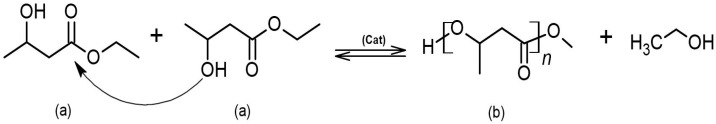
Reaction mechanism of self-polycondensation of (**a**) racemic ethyl 3-hydroxybutyrate to (**b**) a-P3HB.

For the synthesis of a-P3HB, racemic ethyl 3-hydroxybutyrate (20 g, 0.151 mol) with a transesterification catalyst (0.2 g, 1 wt%) was placed in a 100 mL single-neck flask. The flask was connected to a vacuum distillation apparatus and heated to 140 °C at 200 mbar. After one hour, the temperature was gradually increased to 200 °C to enhance the removal of the formed ethanol and to achieve a higher reaction rate and molecular weight. Different catalysts were investigated (Table 1), and the polymerization was confirmed and followed by ethanol formation on FTIR and observing the steam temperature in the distillation apparatus. The product was then analyzed by GPC to determine the resulting molecular weight. Figure 5 illustrates the reaction mechanism of the self-polycondensation of racemic ethyl 3-hydroxybutyrate.

To optimize the reaction parameters and to observe their influence on the resulting molecular weight of a-P3HB as well as the extent of the elimination reaction occurring at high temperatures, statistical experiments were designed and analyzed using the software MODDE 13 from Satorius (Göttingen, Germany). The experiments were carried out with titanium isopropoxide as the catalyst and the reaction parameters were varied between 140–200 °C as an end temperature, 0.05–1 wt% for the catalyst and between 4–8 h of reaction time. The a-P3HB obtained in these experiments was also analyzed by UV–VIS microscopy by using 3-butenoic acid as a standard to determine the double bond content of the side products that could be obtained at high temperature due to the elimination reaction.

**Figure 6:**
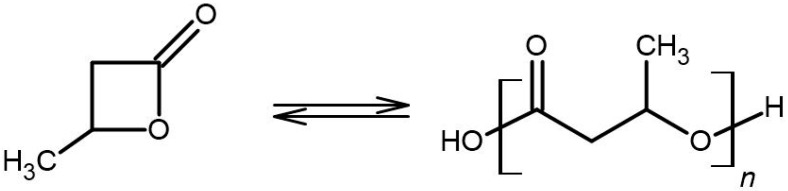
Reaction mechanism of ring-opening polymerization of β-butyrolactone.

In addition, a-P3HB was also synthesized by ring-opening polymerization (ROP) for comparison with the polymer obtained by self-polycondensation. Racemic β-butyrolactone was used for the polymerization reaction, which was conducted at a temperature of 20 °C in anhydrous tetrahydrofuran (THF) solution under argon for 120 h as described in [21] (Figure 6). The monomer concentration was 1.0 mol/L, and the potassium methoxide/18-crown-6 concentration was 0.01 mol/L. The polymer was precipitated in methanol and dried under a vacuum. The progress of the reaction was followed on the FTIR by the shift of the band from β-butyrolactone (1815 cm^−1^) to (1730 cm^−1^) of poly3-hydroxybutyrate.

### 2.3. Fourier Transform Infrared Spectroscopy

Fourier Transform Infrared Spectra were recorded of the racemic ethyl-3-hydroxybutyrate as a reference material, and of the a-P3HB synthesized via self-polycondensation and ROP, as well as of the distillate on a Perkin Elmer FTIR spectrometer (Rodgau, Germany) at room temperature. The samples were analyzed in the range of 650–4000 cm^−1^ and 32 scans were recorded for better accuracy.

### 2.4. Gel Permeation Chromatography

The molecular weight range of the obtained polymers was determined by gel permeation chromatography using an Agilent chromatograph (Waldbronn, Germany) conducted in chloroform and calibrated with polystyrene standards. The GPC was equipped with a UV detector and a differential refractive index detector (1260 infinity) with two styrene-divinylbenzene columns (StyDiViBe-P10E5A-BPT, 300 mm × 4.6 mm), (Applichrom GmbH, Darmstadt, Germany) connected in series with a flow rate of 0.3 mL/min at 40 bar and 30 °C. Samples were prepared by dissolving in chloroform (HPLC grade) with a concentration of 5 mg/mL, and 20 µL of the solution was injected for measurement.

### 2.5. UV–VIS Spectroscopy

UV–VIS absorption spectra of a-P3HB obtained by self-polycondensation of racemic ethy-3-hydroxybutyrate were recorded on a Hach UV–VIS spectrometer (Düsseldorf, Germany). Samples of a-P3HB of known concentration were dissolved in chloroform, and 3-butenoic acid was used as a standard to determine the double bond content in the samples, showing the maximum of absorption at 240 nm. The deviation in the quantification of the results was within ±0.0010%.

## 3. Results and Discussion

### 3.1. Synthesis of a-P3HB by Self-Polycondensation and ROP

**Figure 7:**
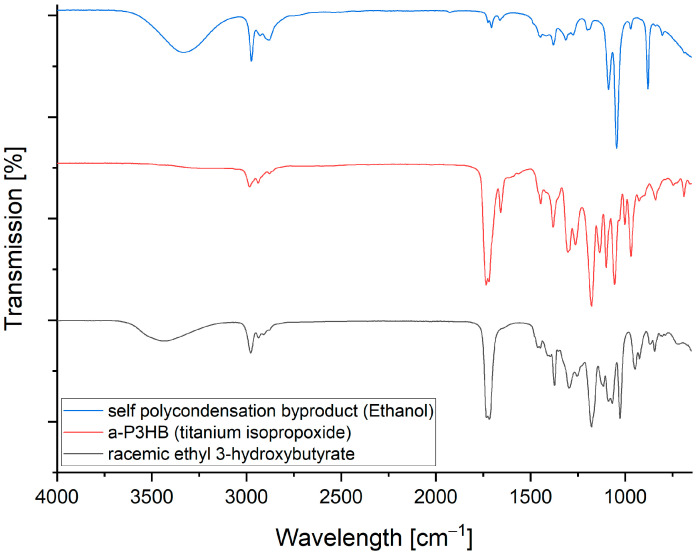
FTIR spectrum of racemic ethyl 3-hydroxybutyrate (black), a-P3HB (red) and the formed ethanol of the self-polycondensation (blue).

The formation of ethanol and the increase in molecular weight observed by GPC were evidence that the self-polycondensation of the racemic ethyl-3-hydroxybutyrate was successful. Figure 7 shows the FTIR spectra of the educt ethyl 3-hydroxybutyrate and the obtained a-P3HB, where we can observe the decrease in the hydroxyl group peak at 3400 cm^−1^ due to the progress of the polymerization, and the distillate which corresponds to the spectrum of ethanol.

**Figure 8:**
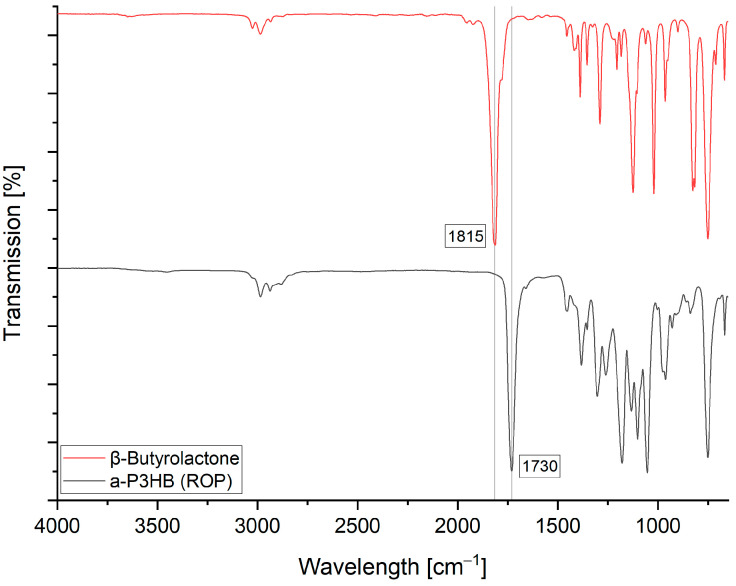
FTIR spectrum of b-butyrolactone (red) and the formed a-P3HB by the ring-opening polymerization (black).

The ROP could also be confirmed on FTIR by shifting the characteristic band from β-butyrolactone (1815 cm^−1^) to (1730 cm^−1^) of poly-3-hydroxybutyrate (Figure 8).

### 3.2. Screening Tests with Different Catalysts

The catalyst screening process in this study revealed intriguing insights into the synthesis of atactic poly-3-hydroxybutyrate through self-polycondensation. Different catalysts exhibited varying degrees of effectiveness, influencing the molecular weight and range of the resulting polymers. This highlights the potential of specific catalysts to play a pivotal role in tailoring the properties of a-P3HB.

The screening tests showed six of ten tested catalysts, where the self-polycondensation was successful. Although the other four catalysts have been reported in the literature as suitable for the synthesis of polyesters through polycondensation, they were not successful in our tests. This could be due to differences in their reactivity, compatibility, or the specific reaction parameters used. Table 1 shows the molecular weight as well as the molecular weight range of the polycondensates determined by GPC and the result of the respective experiment as successful or unsuccessful. While the tin catalysts like tin (II) 2-ethylhexanoate, tin chloride and DBTL were able to promote the reaction successfully, the molecular weight of a-P3HB obtained was relatively low and not as high as that obtained by ROP. Self-polycondensation also took place with Iron (III) Chloride hexahydrate as a catalyst, but the polymer obtained had a molecular weight similar to that obtained with tin catalysts of about 1000 g/mol. Sulfuric acid was also successful in promoting the reaction and in obtaining a polymer with a slightly higher molecular weight than the latter of 1253 g/mol. a-P3HB with the highest molecular weight obtained by self-polycondensation of 2352 g/mol was obtained using titanium isopropoxide as a catalyst. The molecular weight range of this polymer, between 31,021 and 694 g/mol, is even higher than that of a-P3HB obtained by ROP in this work. Additionally, it is important to mention that a-P3HB with a higher molecular weight can be also obtained by ROP using distannoxane catalysts as described in reference [22].

**Figure 9:**
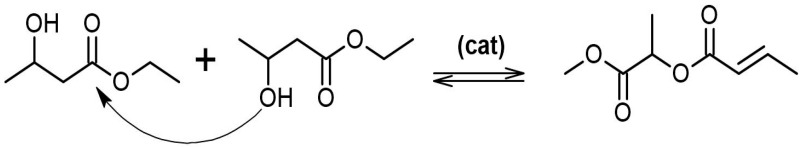
Product of the elimination reaction.

**Figure 10:**
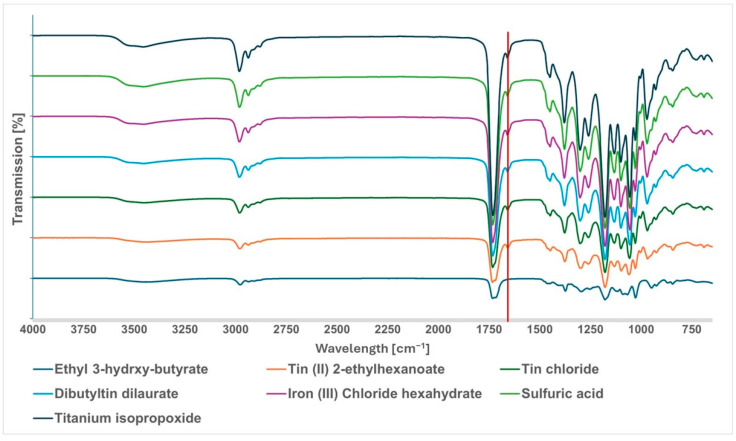
FTIR spectra of a-PHB synthesized by self-polycondensation with various catalysts.

**Figure 11:**
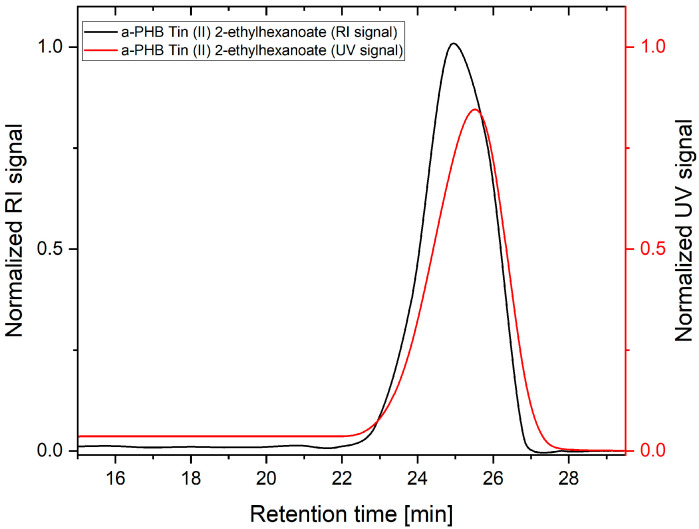
GPC result of a-PHB from self-polycondensation. (RI signal in black, UV signal in red).

Since the reaction temperature had to be gradually increased during the self-polycondensation to achieve a high conversion and to remove the formed ethanol from the reaction mixture, high temperatures also led to elimination reactions, so that some of the formed polymer chains had terminal double bonds and thus could not polymerize further, as shown in Figure 9. Therefore, the elimination reaction as a competitive reaction limits the molecular weight that can be achieved in this synthesis. Besides the elimination reaction, the steric hindrance of the hydroxy group of the monomer is another reason for the difficulty in achieving a high molecular weight. The double bond formed by the elimination reaction could be detected by the new bands in the FTIR spectrum at 1650 cm^−1^ and in the fingerprint region at 970 cm^−1^ (Figure 10). The polymer chains with double bonds resulting from the elimination reaction were also detected by the UV detector of the GPC (Figure 11), and the double bond content was determined by UV–VIS spectroscopy.

### 3.3. Optimizing the Reaction Parameters

Optimizing the reaction parameters proved crucial in influencing the molecular weight of a-P3HB. Insights from statistical experiments emphasized the delicate balance required in reaction conditions for optimal molecular weight outcomes.

**Figure 12:**
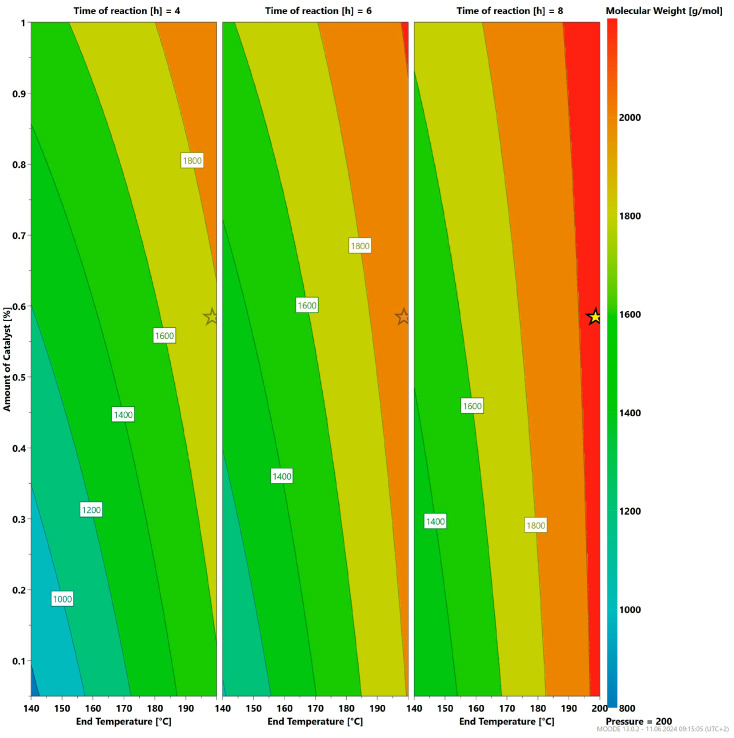
Influence of the reaction parameter on the resulting molecular weight; the yellow star indicates the calculated optimal reaction parameters from the DOE model.

**Figure 13:**
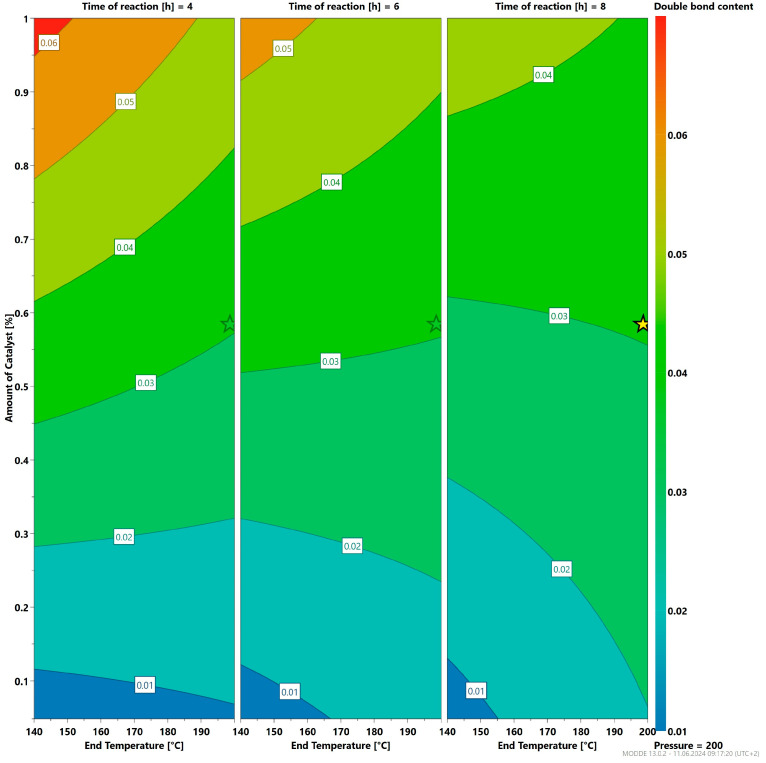
Influence of the amount of catalyst on the double bond content; the yellow star indicates the calculated optimal amount of catalyst from the DOE model.

The results of statistical experiments showed that elevated temperatures of 200 °C at 200 mbar are necessary to enhance the molecular weight growth. a-P3HB with the highest molecular weight in these experiments was obtained after six to eight hours of reaction (Figure 12). The amount of catalyst was also varied between 0.05 wt% and 1 wt% to determine its effect on the polymerization process. The results calculated by Modde^TM^, as shown in Figure 12 and Figure 13, indicate that 0.5 wt% of catalyst is sufficient for the self-polycondensation to occur, and the polymer achieved the highest molecular weight. Additionally, this concentration also results in a low double bond content, which is desirable in this synthesis. Another crucial result of these experiments is that a higher amount of catalyst of 1 wt% even at the low temperature of 140 °C leads to a higher extent of elimination reaction, and thus a higher double bond content (Figure 13).

### 3.4. LCA Analysis of a-P3HB Chemical Synthesis

The aim of the LCA analysis is to assess the environmental impact of the chemical synthesized a-P3HB and compare it with the biotechnological process as well as the conventional petrochemical polymers, such as polyethylene and polypropylene. Therefore, all the available data of the chemical synthesis of a-PHB by self-polycondensation and ROP, including used chemicals and energy usage, were collected. The collected data were analyzed with the software Umberto of IPoint-systems 10 (Hamburg, Germany) using the ecoinvent 3.9.1 database.

According to the findings from the life cycle assessment (Table 2), the chemical synthesis of a-P3HB by self-polycondensation exhibits the lowest CO_2_ emissions of 1.7 kg CO_2_/kg polymer, as well as the lowest energy consumption of 12.7 MJ/kg when compared to the biogenesis, ROP, and polyolefins production. It is important to note that these conclusions are drawn from a laboratory-scale analysis, and there is an expectation that values would improve in industrial-scale a-P3HB production. The low energy consumption in the chemical synthesis results from the straightforward one-step process and relatively short reaction time. On the other hand, the synthesis of a-P3HB through ring-opening polymerization lasts for 120 h, resulting in an elevated energy consumption and subsequent high CO_2_ emissions. This renders the ROP synthesis economically unfavorable.

## 4. Conclusions

In this work, we were able to synthesize the atactic poly-3-hydroxybutyrate by self-polycondensation of racemic ethyl 3-hydroxybutyrate. The reaction was monitored by FTIR and approved by the increasing of molecular weight and the formation of ethanol as a by-product of the polycondensation reaction. The molecular weight of the obtained products was determined by GPC and the double bond content resulting from the elimination reaction at an elevated temperature was determined by UV–VIS spectroscopy. Screening tests showed six catalysts that were able to promote the reaction successfully. The highest molecular weight obtained by self-polycondensation of 2352 g/mol was reached with titanium isopropoxide as a catalyst. The molecular weight range of this polymer, between 31,021 and 694 g/mol, was also higher than that of the product obtained in this study by the ring-opening polymerization of β-butyrolactone (10,061–147 g/mol), which is the most reported method in the literature for the chemical synthesis of a-P3HB. The reaction parameters were also optimized by statistical experiments, showing that 200 °C and 8 h with 0.5 wt% of catalyst are required for a high molecular weight. The successful synthesis of a-P3HB through self-polycondensation, coupled with the catalyst screening and optimization insights, opens avenues for further exploration and application. The ability to reduce the crystallinity of i-P3HB and enhance processability through a-P3HB blending or copolymerization underscores its potential in diverse industrial sectors.

## Figures and Tables

**Table 1:** Molecular weight of a-P3HB obtained by self-polycondensation of racemic ethyl-3-hydroxybutyrate and ring-opening polymerization determined by GPC.

Catalyst	Result	Mw (g/mol)	Mw Range (g/mol)	PDI	DBC (%)
Tin (II) 2-ethylhexanoate	successful	906	(3268–268)	1.2	0.0197 (±0.0002)
Tin chloride	successful	696	(2499–263)	1.1	0.0436 (±0.0012)
Dibutyltin dilaurate	successful	1067	(6131–263)	1.3	0.0198 (±0.0002)
Zn acetate	unsuccessful	-			
Sodium methoxide	unsuccessful	-			
Aluminum (III) chloride	unsuccessful	-			
Iron (III) Chloride hexahydrate	successful	908	(6908–238)	1.3	0.1008 (±0.0023)
p-toluene sulfonic acid	unsuccessful	-			
Sulfuric acid ^1^	successful	1253	(9102–343)	1.8	0.0036 (±0.0002)
Titanium isopropoxide	successful	2352	(31,021–694)	1.7	0.0674 (±0.0012)
KOCH3/18-crown-6	successful	3443 ^2^	(10,061–147)	1.5	0.0343 (±0.0007)

^1^ the concentration of the sulfuric acid solution was 1 mol/L. ^2^ obtained by ring-opening polymerization.

**Table 2:** Results of LCA Analysis of a-PHB and petrochemical polymers.

	Polymer	CO_2_ Emissions	Energy Consumption	References
		kg CO_2_/kg Polymer	MJ/kg Polymer	
PHA	P3HB (fermentation)	2.6	44.7	[23]
	a-P3HB (self-polycondensation)	1.7	12.7	
	a-PHB (ROP)	13.7	22.5	
Polyolefins	Polypropylene (PP)	3.4	85.9	[24]
	High density polyethylene (HDPE)	2.5	73.7	[24]
	Low density polyethylene (LDPE)	3.0	81.8	[24]

## Data Availability

Data are contained within the article.
